# Disability after non‐cardiac surgery – patient‐centred outcomes and resource utilisation after non‐cardiac surgery (PACORUS): a prospective multicentre cohort study*

**DOI:** 10.1111/anae.70248

**Published:** 2026-06-11

**Authors:** Alexandra Stroda, Jan Larmann, Andrea Icks, Mark Coburn, Marc Moritz Berger, Alexander Mathes, Robert Werdehausen, Sarah Thaler, Sebastian Ziemann, Claudia Neumann, Sebastian Roth, René M'Pembele, Theresa Tenge, Celine Bußhoff, Madeleine Rallis, Giovanna Lurati Buse, the PACORUS‐D Investigators, Onur Aydemir, Tabea Schüssler, Linda Grüßer, Ana Kowark, Andrea Kunsorg, Maria Wittmann, Franziska Torabi, Jakob Labus, Jens Brands, Thorsten Brenner, Theresa Berger, Sarah Dehne, Melanie Etheredge, Markus A. Weigand, Simon Schäfer, Christian Schlegel, Antje Seidel

**Affiliations:** ^1^ Department of Anaesthesiology, Medical Faculty University of Duesseldorf Duesseldorf Germany; ^2^ Department of Anaesthesiology, Medical Faculty RWTH Aachen University Aachen Germany; ^3^ Department of Anaesthesiology, Medical Faculty University of Heidelberg Heidelberg Germany; ^4^ Centre for Health and Society, Institute for Health Services Research and Health Economics Heinrich‐Heine‐University Duesseldorf Germany; ^5^ German Diabetes Center, Leibniz Center for Diabetes Research Institute for Health Services Research and Health Economics Duesseldorf Germany; ^6^ Department of Anaesthesiology and Intensive Care Medicine University Hospital Bonn Bonn Germany; ^7^ Department of Anaesthesiology, Intensive Care Medicine, Emergency Medicine and Pain Medicine RKH Hospital Ludwigsburg Ludwigsburg Germany; ^8^ Department of Anaesthesiology and Intensive Care Medicine University Hospital Essen, University Duisburg‐Essen Essen Germany; ^9^ Department of Anaesthesiology and Intensive Care Medicine University of Cologne, Faculty of Medicine and University Hospital Cologne Germany; ^10^ Department of Anaesthesiology and Intensive Care, Medical Faculty University of Magdeburg Magdeburg Germany; ^11^ Department of Anaesthesiology and Intensive Care Medicine University Hospital Leipzig Leipzig Germany; ^12^ Department of Anaesthesiology Ludwig‐Maximilians‐University Munich Munich Germany

**Keywords:** patient‐centred outcomes, peri‐operative medicine, postoperative outcome measures, WHODAS 2.0, disability

## Abstract

**Introduction:**

The Standardised Endpoints in Perioperative Medicine Initiative recommends the 12‐item WHO Disability Assessment Schedule (WHODAS) 2.0 to measure postoperative functional impairment. However, evidence describing long‐term postoperative disability, particularly at 1 year, remains limited. This study aimed to: describe disability up to 1 year after non‐cardiac, non‐neurosurgical procedures; examine associations between periprocedural risk, postoperative complications and new‐onset disability; and evaluate associations between social support, delirium, and frailty and postoperative disability.

**Methods:**

We conducted a prospective, multicentre cohort study. The primary endpoint was new onset clinically significant disability (an increase in WHODAS 2.0 score ≥ 5% points from baseline to a final score ≥ 35%) or death at 1 year. Independent variables included: periprocedural risk; postoperative complications; frailty; and patient‐reported social support.

**Results:**

In total, 1081 patients were included: 726 (67%) male, median (IQR) [range] age 67 (60–75 [50–101]) y. New onset clinically significant disability was present in 139/1081 (12.9%) patients at 1 year and in 238/1066 (22.3%) patients at 30 days. Periprocedural risk (adjusted odds ratio (aOR) 1.05, 95%CI 1.03–1.07, per percent in American College of Surgeons National Surgical Quality Improvement Program score) and postoperative complications of all grades were associated with 1‐year new onset clinically significant disability. New onset (1 year) clinically significant disability was not associated with social support: OR 0.87, 95%CI 0.54–1.39 for moderate; and OR 0.76, 95%CI 0.47–1.24 for strong social support. Frailty was strongly associated with new onset clinically significant disability after 1 year (aOR 3.97, 95%CI 2.54–6.21).

**Discussion:**

One year after non‐cardiac surgery, 13% of surviving patients experienced a new onset clinically significant disability. Postoperative complications, even those of low severity, were associated with new onset postoperative disability.

## Plain Language Summary

**What we did:**

We followed a large group of people who had surgery that was not on the brain or heart. We checked how well they were able to do everyday activities before surgery, then again after 30 days and after 1 year. We also looked at things like how risky their surgery was, if they had problems after surgery, how strong or frail they were and whether they had support from others.

**Why did we do it:**

We wanted to understand how surgery affects people in the long term, especially whether they develop new disabilities that make daily life harder. This helps doctors know who might need extra care and support after surgery.

**What we found:**

We found that about 1 in 8 people had new problems with daily activities one year after surgery. More people had problems soon after surgery, but some improved over time. People who had higher risk for surgery, had complications after surgery, or were already frail were more likely to have long‐term disability. Having social support did not seem to make a big difference in this study. This shows that preventing complications and supporting frail patients is very important for recovery.

## Introduction

In Europe, more than 6500 surgical procedures per 100,000 individuals are conducted annually [1]. As peri‐operative mortality rates have decreased due to improved peri‐operative safety, greater attention has been directed towards functional outcomes. These outcomes focus on patient experience and the impact of surgery on daily life, as highlighted in the section on patient‐centred outcomes by the Standardised Endpoints in Perioperative Medicine (StEP) Initiative [2].

The 12‐item WHO Disability Assessment Schedule (WHODAS) 2.0 is recommended by the StEP Initiative as the preferred instrument for assessing postoperative functional disability, ideally performed at 12 months [2]. The WHODAS 2.0 covers six domains: cognition; mobility; self‐care; interpersonal relationships; work/household roles; and participation in society [3]. Initial validation in a multicentre surgical cohort (n = 510) showed good internal consistency, with 13% of patients having new disability at 12 months [4]. Despite this validation, data on the incidence, domain‐specific patterns, and risk factors for disability remain limited, particularly with respect to the role of postoperative complications, a potentially modifiable factor. In the initial validation study, patients with new disability had longer hospital stays and more complications, though these findings were based on unadjusted analyses [4].

In 2020, Shulman et al. proposed surgery‐specific cut‐offs for new onset clinically significant disability based on data from one large randomised controlled trial (n = 2983) [5] and two cohorts (n = 510 and n = 868) [6]. Using these cut‐offs, 10% of patients had clinically significant disability and 6% developed new disability at 6 months; however, these estimates may have limited generalisability due to the inclusion of patients having cardiac and neurosurgery, who typically face different surgical burdens and structured rehabilitation. More recently, in 2025, Wijeysundera et al. provided evidence on disability trajectories up to 1 year after elective non‐cardiac surgery [7]. Whilst this study provided detailed analyses of pre‐operative predictors of postoperative disability, the contribution of postoperative complications was not addressed.

The primary aims of this present study were: to describe the incidence of new onset clinically significant disability at 30 days and 1 year after non‐cardiac surgery; to examine associations between periprocedural risk, postoperative complications and new onset disability; and to evaluate associations of delirium and frailty (in patients aged ≥ 65 y) with disability. Social isolation has been linked to adverse outcomes [8]. Accordingly, a secondary aim of this study was to assess the association between patient‐reported social support and the incidence of new onset clinically significant disability at 30 days and 1 year after surgery.

## Methods

We conducted a prospective multicentre cohort study at eight German University hospitals from April 2021 to July 2022. The study adhered to the Declaration of Helsinki and General Data Protection Regulation (EU) 2016/679. Ethical approval and written informed consent were obtained at all sites. Centres opted into participating in a nested cohort assessing delirium using the Confusion Assessment Method (CAM), requiring separate consent. Reporting of this study follows STROBE guidelines.

Patients aged ≥ 50 y having non‐cardiac, non‐neurosurgical (thoracic, vascular, abdominal, urinary tract, or orthopaedic/trauma) inpatient surgery were included. Emergent and weekend cases were approached on the next working day after surgery. Patients who were unwilling or unable to provide informed consent; had their procedure cancelled; had procedures using local anaesthesia or sedation; had more than one surgical procedure in the 7 days before enrolment; were unable to complete the WHODAS 2.0 questionnaire (e.g. language or literacy barriers); and were previously enrolled in this study, were not studied.

The primary endpoint was new onset clinically significant disability (defined as an increase in WHODAS 2.0 score of at least 5% points compared with baseline and reaching a final WHODAS 2.0 score of at least 35% [6]) or death at 1 year. Patients completed the 12‐item WHODAS 2.0 questionnaires at: baseline (pre‐operatively); 30 days postoperatively; and 1 year after surgery. Baseline and in‐hospital assessments were done in person; 30‐day and 1‐year follow‐ups were conducted by trained researchers via telephone or postal mail [3]. Scores for 12 items (0–4 scale) were summed (0–48) and converted to a 0–100% scale, where 0% indicates no disability and 100% complete disability [3]. Following Shulman et al., disability was categorised as none (≤ 15%); mild (16–34%); significant (≥ 35%); and new onset clinically significant disability (≥ 35% plus ≥ 5% point increase) [6]. Secondary endpoints consisted of: new onset clinically significant disability (as defined above) or death at 30 days; clinically relevant disability at 30 days and 1 year (defined as 12‐item WHODAS 2.0 > 35% [6]); and trends in continuous WHODAS 2.0 score at 30 days and 1 year (for this analysis, only survivors at 30 days and 1 year were included).

We investigated the contribution of pre‐, intra‐ and postoperative components to the outcomes mentioned above. The main exposures were periprocedural risk and postoperative complications. Pre‐operative risk was estimated using the American College of Surgeons National Surgical Quality Improvement Program (ACS NSQIP) surgical risk score for complications [9], calculated from patient data by trained personnel via the online ACS NSQIP calculator, reflecting pre‐ and intra‐operative components. Postoperative components comprised in‐hospital postoperative complications which were extracted from medical charts at discharge and categorised according to the Clavien‐Dindo classification (grades 1–5) [10, 11, 12].

In the primary analysis, complications were modelled as an ordinal variable (with grades 4a and 4b pooled due to the limited number in each category). In a sensitivity analysis, complications were additionally modelled as a binary variable (complication yes/no). In patients aged ≥ 65 y, frailty was assessed using the Clinical Frailty Scale (CFS), with scores ≥ 4 indicating frailty [13, 14, 15, 16, 17]. All frailty assessments were conducted either by a study clinician or by a trained clinical research assistant during the patient's visit to the pre‐operative assessment unit, 1–5 days before surgery.

Social support was measured pre‐operatively using the Oslo Social Support Scale (OSSS‐3) [18] by trained research personnel. This consists of three questions with a final score ranging from 3 to 14 points: 3–8 points indicate poor social support; 9–11 points, moderate social support; and 12–14 points, strong social support [18].

Additional model variables included baseline 12‐item WHODAS 2.0 scores and a cancer diagnosis as indication for surgery. Age and sex were not included as separate covariates because they are incorporated within the ACS NSQIP risk score.

We assumed that at 1 year, 20% of the patients would have a new onset clinically significant disability [6]. Based on 10–12 events per variable [19], the planned sample size of 1200 patients (assuming ≤ 15% [4, 6] non‐response at 1 year) would allow for a robust logistic regression model with up to 20 variables. In the event, the model included four variables, and the analysis was based on 31 events per variable.

Statistical analysis followed a predefined plan and was performed using SPSS (version 29.0, SPSS, Chicago, IL, USA), with significance set at a 2‐tailed p < 0.05. Missing data were not imputed (complete case approach). Descriptive analyses included Sankey diagrams to illustrate disability progression up to 1 year postoperatively, presented overall and stratified by surgical category and ACS NSQIP quartiles. Multivariable logistic regression with forced entry of the independent variables was used to examine associations between periprocedural risk (ACS NSQIP, continuous) and postoperative complications (Clavien‐Dindo, ordinal) with binary outcomes. The main model performance in terms of discrimination was evaluated using the area under the receiver operating characteristic curve (AUROC). Calibration was evaluated with the Hosmer‐Lemeshow goodness‐of‐fit test and a calibration plot. Multivariate quantile regression was used to analyse continuous WHODAS 2.0 scores. Frailty was added in additional logistic and quantile regression models analysing the subsample of patients aged ≥ 65 y. Social support was analysed using univariate logistic regression and included in the main model if significantly associated. In addition, we tested for effect modification by sex and daily physical activity by incorporating interaction terms into the multivariable logistic regression model.

We conducted the following predefined sensitivity analyses: new onset clinically significant disability at 30 days and 1 year in survivors only; disability (defined at 12‐item WHODAS 2.0 ≥ 25% as proposed for the general setting); periprocedural risk estimated using the Charlson Comorbidity Index (CCI, 0–37 points) [20, 21] and the Preoperative Score to Predict Postoperative Mortality (POSPOM, 0–50 points) [22] instead of the ACS NSQIP risk calculators; complications as binary events instead of ordinal definition (Clavien‐Dindo classification); and as an alternative definition for social support, we used the presence of at least one independent household member.

During the review process, the following sensitivity analyses were added: alternative definition of new disability defined as 5% points difference in WHODAS 2.0 at 1 year (and 30 days) compared with baseline; and we conducted two ordinal logistic regression models for the association of periprocedural risk and complications with disability, categorised as no disability, new onset clinically significant disability and dead in order to address the potential differences in significant disability and death. In the second ordinal regression, disability was categorised as no disability (WHODAS 2.0 score < 16%); mild (WHODAS 2.0 score 16–34%); significant (WHODAS 2.0 score ≥ 35%); and dead.

## Results

The analysis included 1081 patients with complete 1‐year follow‐up, of whom 726 (67%) were male (Fig. 1). Median (IQR [range]) age was 67 (60–75 [50–101]) y and duration of hospital stay was 6 (3–10 [0–125]) days. Baseline characteristics are presented in Table 1 and online Supporting Information Table S1. The characteristics of patients without any disability at 1 year and patients lost to follow up at 1 year are show in online Supporting Information Table S2. Due to 4.5% missing 30‐day WHODAS 2.0 data (51/1117), 1066 patients were included in the 30‐day analysis (Fig. 1).

**Figure 1:**
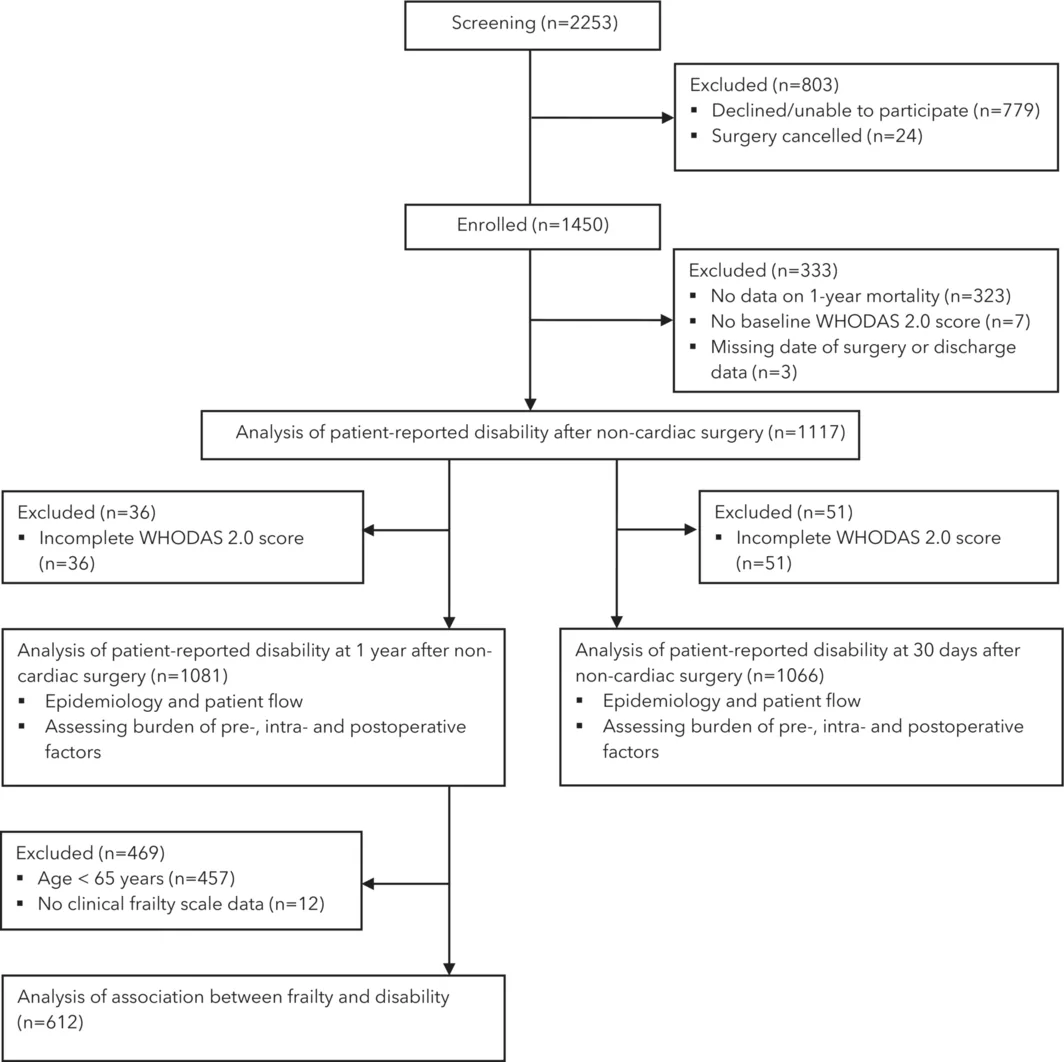
Study flow chart.

**Table 1:** Summary of baseline patient characteristics (full data available in online Supporting Information Table S1). Values are median (IQR [range]) or number (proportion).

	Full dataset	Patients with new onset clinically significant disability or dead at 1 year	Patients with no new onset clinically significant disability and alive at 1 year
	n = 1081	n = 218	n = 863
Age; y	67 (60–75 [50–101])	71 (62–78 [50–94])	66 (59–74 [50–101])
Sex; male	726 (67.1%)	130 (59.6%)	596 (69.1%)
Oslo Social Support‐3 scale	n = 1080	n = 217	n = 863
Poor social support	121 (11.2%)	28 (12.9%)	93 (10.8%)
Moderate social sport	493 (45.6%)	102 (47.0%)	391 (45.3%)
Strong social support	466 (43.2%)	87 (40.1%)	379 (43.9%)
Pre‐operative Clinical Frailty Scale	n = 834	n = 177	n = 657
1: Very fit	90 (10.8%)	6 (3.4%)	84 (12.7%)
2: Well	315 (37.8%)	38 (21.5%)	277 (42.2%)
3: Managing well	229 (27.5%)	51 (28.8%)	178 (27.1%)
4: Vulnerable	98 (11.7%)	41 (23.2%)	57 (8.7%)
5: Mildly frail	43 (5.2%)	16 (9.0%)	27 (4.2%)
6: Moderately frail	32 (3.8%)	14 (7.9%)	18 (2.7%)
7: Severely frail	23 (2.7%)	11 (6.2%)	12 (1.8%)
8: Very severely frail	4 (0.5%)	0	4 (0.6%)
Pre‐operative functional status	n = 1080	n = 218	n = 863
Fully independent	905 (83.8%)	144 (66.1%)	761 (88.2%)
Partially dependent	164 (15.2%)	69 (31.7%)	95 (11.0%)
Fully dependent	12 (1.0%)	5 (2.3%)	7 (0.8%)
Baseline WHODAS 2.0 score	10.4 (4.2–27.1 [0.0–85.4])	23 (10.4–40 [0.0–72.9])	8.3 (4.2–23 [0.0–85.4])
Daily physical activities	n = 896	n = 173	n = 723
Inactive	173 (19.3%)	42 (24.3%)	131 (18.2%)
Regularly low level	199 (22.2%)	55 (31.8%)	144 (19.9%)
20–60 min per week	160 (17.9%)	30 (17.3%)	130 (17.9%)
1–3 h per week	177 (19.8%)	24 (13.9%)	153 (21.2%)
> 3 h per week	187 (20.8%)	22 (12.7%)	165 (22.8%)

WHODAS, World Health Organization Disability assessment schedule.

At 1 year, 218/1081 (20%) patients met the primary outcome, of whom 79/1081 (7.3%) had died. Among the 1002 survivors, 139 (13.9%) developed new onset clinically significant disability at 1 year (including 86/682 (12.6%) of surviving men and 53/320 (16.5%) of surviving women). Figure 2 illustrates disability progression up to 1 year postoperatively. Figure 3 presents domain‐specific WHODAS 2.0 scores at baseline and at 1 year among patients with new onset disability, with sex‐stratified results shown in online Supporting Information Figure S1. At 30 days, 252/1066 (23.6%) patients met the primary outcome of whom 14/1066 (1.3%) had died. New onset clinically significant disability at 30 days was present in 238/1052 (22.6%) survivors (including 129/706 (18.2%) of surviving men and 109/346 (31.5%) of surviving women) (online Supporting Information Table S3).

**Figure 2:**
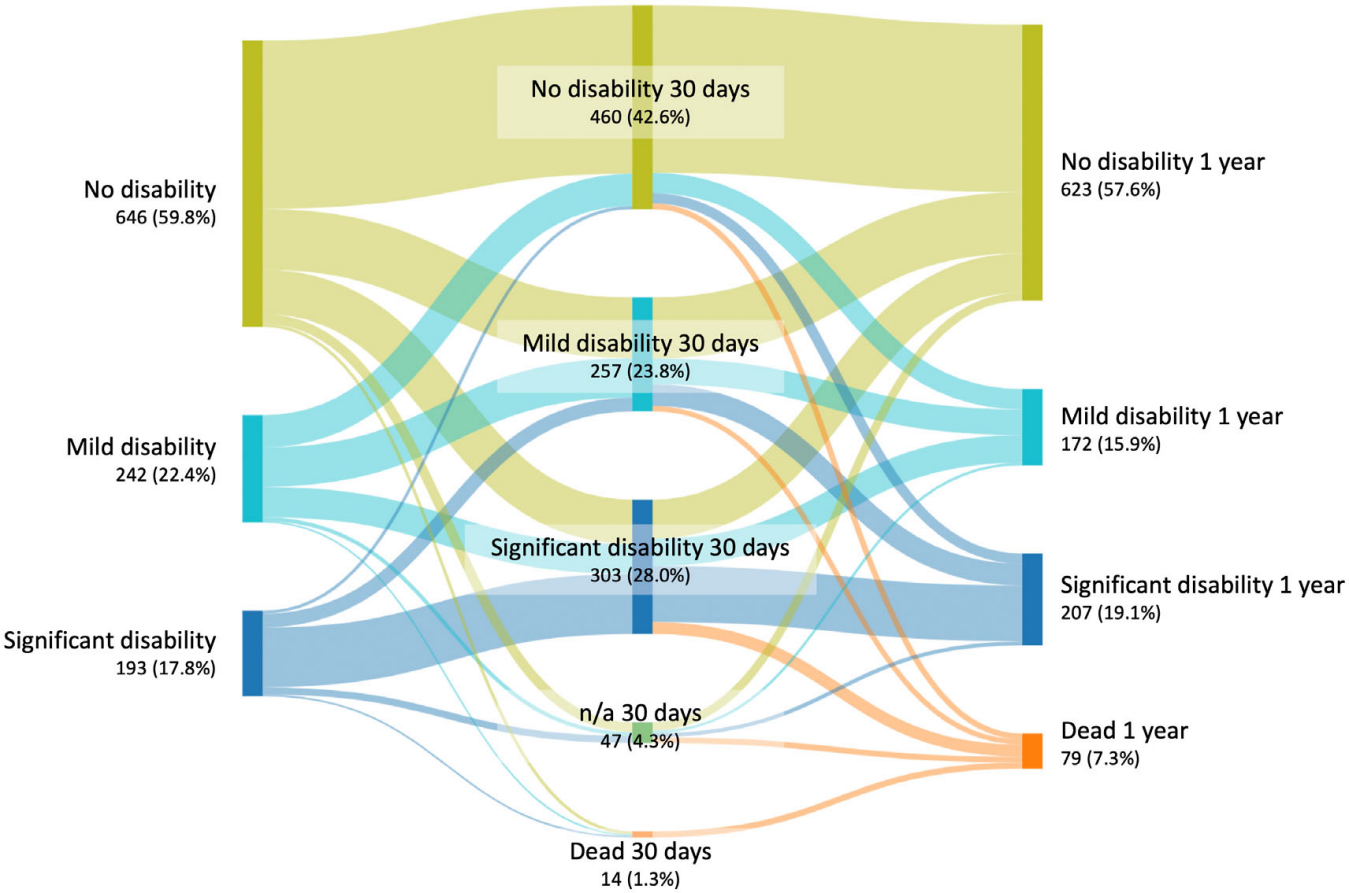
Patient‐reported disability severity from pre‐operative status to 1 year after non‐cardiac surgery (n = 1081). Disability defined by WHODAS 2.0 score: no disability 0–15%; mild disability 16–34%; significant disability ≥ 35%. n/a, not applicable.

**Figure 3:**
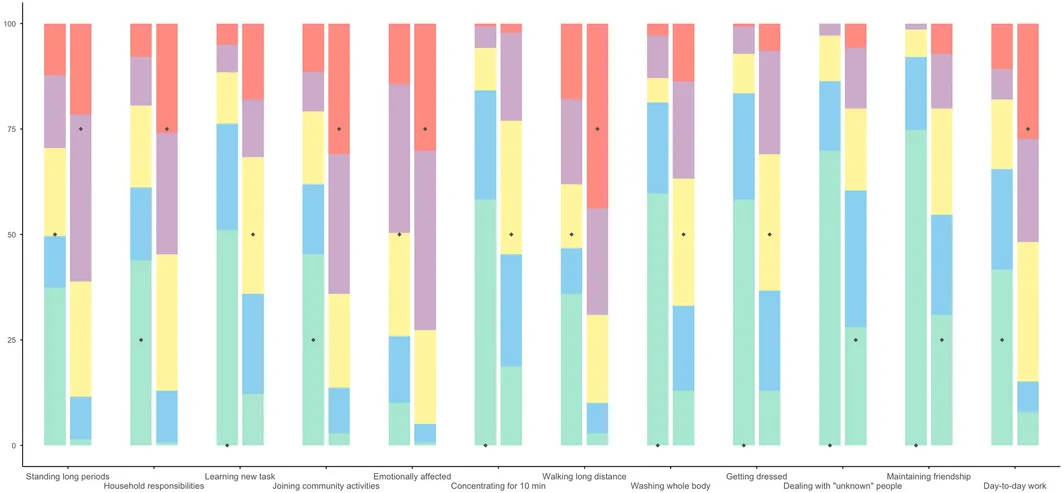
Distribution of the answers to the single items at baseline and at 1 year in the patients with new onset disability after 1 year: 0, none (green); 1, mild (blue); 2, moderate (yellow); 3, severe (purple); 4, extreme or cannot do (red). Black diamonds, median of the responses.

Table 2 shows the periprocedural scores and surgical information of full cohort and patients with and without primary outcome. The surgical category of patients was classified using the definitions of the POSPOM [22]. Distribution of surgical categories is available in online Supporting Information Table S4. Incidence and trajectories of new onset clinically significant disability and death at 30 days and 1 year stratified by surgical category are detailed in online Supporting Information Table S5 and Figure S2. Detailed postoperative information is shown in Table 3. Patient‐reported disability by ACS NSQIP score quartiles and by postoperative complications, respectively, are available in online Supporting Information Figure S3 and Table S6. Detailed information on the distribution of postoperative complications in patients with and without the primary outcome, as well as the relative risk for the occurrence of new onset clinically significant disability or death at 1 year, is available in online Supporting Information Table S7.

**Table 2:** Periprocedural scores and surgical information. Values are number (proportion) or median (IQR [range]). Detailed information on surgical category is in online Supporting Information Table S4.

	Full dataset	Patients with new onset clinically significant disability or dead at 1 year	Patients without new onset clinically significant disability and alive at 1 year
	n = 1081	n = 218	n = 863
ACS NSQIP complication	7 (4.5–13.3 [0–52.2])	10.9 (6.3–18.9 [1.4–52.2])	6.4 (4.1–11.3 [0–48.0])
ACS NSQIP death	0.2 (0.1–1.0 [0–46.6])	0.8 (0.2–3.0 [0–46.6])	0.2 (0–0.8 [0–27.6])
POSPOM (points)	26 (22–30 [7–44])	29 (26–32 [14–44])	25 (21–29 [7–40])
Charlson Comorbidity Index (points)	4 (3–6 [1–13])	6 (4–8 [1–13])	4 (3–6 [1–13])
Emergency case	74 (6.8%)	19 (8.7%)	55 (6.4%)
Major surgery (n = 1076)	577 (53.4%)	128 (58.7%)	449 (52.0%)
Cancer surgery	384 (355%)	90 (41.3%)	294 (341%)

ACS NSQIP, American College of Surgeon National Surgical Quality Improvement Program; POSPOM, Preoperative Score to Predict Postoperative Mortality.

**Table 3:** Postoperative information. Values are number (proportion) or median (IQR [range]). Detailed information on binary adverse postoperative events is in online Supporting Information Table S7.

	Full dataset	Patients with new onset clinically significant disability or dead at 1 year	Patients without new onset clinically significant disability and alive at 1 year
	n = 1081	n = 218	n = 863
ICU admission	159 (14.7%)	64 (29.4%)	95 (11.0%)
Unplanned ICU admission	32/159 (20.1%)	18/64 (28.1%)	14/95 (14.7%)
In‐hospital postoperative complication	308 (28.5%)	107 (49.1%)	201 (23.3%)
Clavien Dindo Grade			
1	104 (33.7%)	27 (25.2%)	77 (38.3%)
2	94 (30.5%)	27 (25.2%)	67 (33.4%)
3a	32 (10.4%)	11 (10.2%)	21 (10.4%)
3b	39 (12.8%)	14 (10.3%)	25 (12.5%)
4a	18 (5.8%)	9 (8.4%)	9 (4.5%)
4b	3 (1.0%)	1 (0.9%)	2 (0.9%)
5	18 (5.8%)	18 (16.8%)	n/a
In‐hospital death	18 (1.7%)	18 (8%)	n/a
30‐day mortality	14 (1.3%)	14 (6.4%)	n/a
1‐year mortality	79 (7.3%)	79 (36%)	n/a
WHDOAS‐2.0 score; %			
30 days	18.8 (6.3–39.6 [0–100]) (n = 1034)	43.8 (23–64.6 [0–100]) (n = 199)	14.6 (4.2–33.3 [0–100]) (n = 835)
1 year	8.3 (2.1–29.2 [0–100])	52.1 (41.7–70.8 [35.4–100])	6.3 (2.1–16.7 [0–83.3])

WHODAS, World Health Organization Disability assessment schedule; n/a, not applicable.

Higher periprocedural risk was associated with new‐onset disability or death at 1 year (aOR 1.05, 95%CI 1.03–1.07). Postoperative complications of any severity were also associated with new onset disability at 1 year (Table 4). The model showed modest discrimination, with an AUROC of 0.71 (95%CI 0.67–0.75). The Hosmer–Lemeshow test did not indicate a lack of fit (p = 0.196), suggesting acceptable model calibration. Visual inspection of the calibration plot suggested reasonable agreement between predicted probabilities and observed outcomes (Fig. 4). The findings for 30‐day new onset disability were consistent, although the association with Clavien‐Dindo grade 2 complications did not reach statistical significance (Table 4). The associations between postoperative complication severity and clinically relevant disability were consistent but not statistically significant across all severity levels (Table 4).

**Table 4:** Multivariate logistic regression model for the association of pre‐operative risk and postoperative complications with primary and secondary endpoint. Values are adjusted odds ratio (95%CI).

	New onset clinically significant disability or death	Clinically relevant disability or death	New onset clinically significant disability or death	Clinically relevant disability or death
	At 1 year		At 30 days	
ACS NSQIP	1.05 (1.03–1.07)	1.04 (1.01–1.06)	1.02 (1.00–1.04)	1.01 (1.01–1.06)
Clavien‐Dindo classification				
1	1.78 (1.08–2.92)	1.91 (1.14–3.21)	2.11 (1.34–3.31)	1.93 (1.17–3.16)
2	1.81 (1.08–3.02)	1.49 (0.84–2.66)	1.45 (0.87–2.42)	1.91 (1.12–3.28)
3a	2.29 (1.04–5.02)	2.65 (1.18–5.95)	2.63 (1.24–5.55)	2.87 (1.29–6.40)
3b	2.57 (1.27–5.21)	2.12 (0.96–4.71)	4.19 (2.11–8.29)	4.36 (1.99–9.53)
4a/b	4.21 (1.72–10.29)	2.19 (0.75–6.42)	3.62 (1.50–8.76)	5.53 (1.84–16.60)
Cancer surgery	1.00 (0.72–1.41)	1.22 (0.84–1.75)	1.06 (0.78–1.46)	1.30 (0.92–1.83)
Baseline WHODAS 2.0 score	n/a	1.07 (1.06–1.08)	n/a	1.07 (1.06–1.08)

ACS NSQIP, American College of Surgeons National Surgical Quality Improvement Program; WHODAS, World Health Organization Disability assessment schedule; n/a, not applicable.

**Figure 4:**
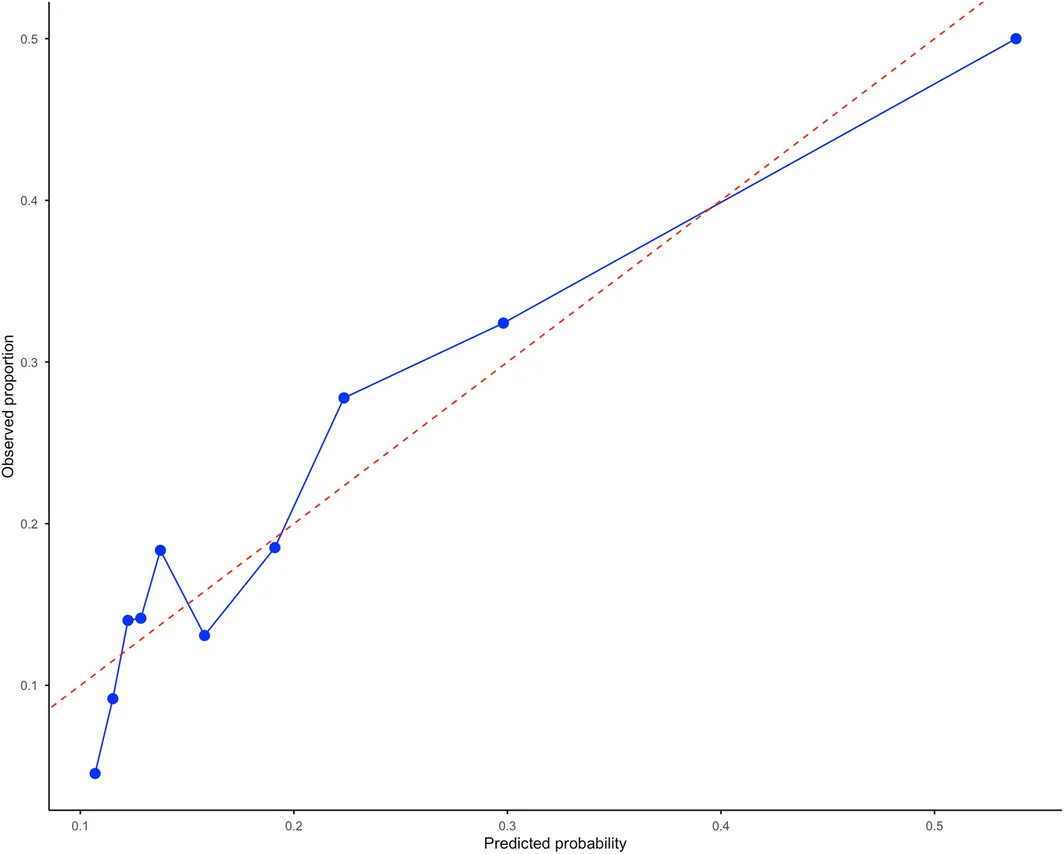
Agreement between predicted probabilities and observed rates of new‐onset clinically significant disability or death for the logistic regression model including ACS NSQIP variables, postoperative complications and cancer surgery. Blue circle, observed vs. red line, predicted.

To assess potential multicollinearity between ACS NSQIP and postoperative complications, collinearity diagnostics were performed using a linear regression model including both predictors. The results indicated no evidence of problematic collinearity (tolerance = 0.873, variance inflation factor = 1.146).

With regard to continuous WHODAS 2.0 scores, the coefficient of multivariate quantile analysis did not suggest a significant association between periprocedural risk and postoperative complications on the first quartile (low score = low disability). In contrast, statistically significant associations were observed at the median and third quartile, indicating greater effects at moderate to higher levels of disability at 1 year (Table 5). Findings for 30‐day continuous WHODAS 2.0 were comparable (online Supporting Information Table S8).

**Table 5:** Multivariate quantile regression analysis for the association of periprocedural risk (ACS NSQIP score for postoperative complications per percent) and postoperative complications with continuous WHODAS 2.0 scores (%) after 1 year.

	0.25‐quantile adjusted regression coefficient (95%CI)	0.50‐quantile adjusted regression coefficient (95%CI)	0.75‐quantile adjusted regression coefficient (95%CI)
ACS NSQIP	0.06 (0.00–0.13)	0.11 (0.10–0.21)	0.42 (0.22–0.63)
Postoperative complications	0.02 (‐0.37–0.41)	1.19 (0.58–1.80)	2.85 (1.59–4.10)
WHODAS 2.0 score at baseline	0.41 (0.38–0.43)	0.83 (0.79–0.87)	0.96 (0.88–1‐04)
Cancer surgery	0.22 (‐0.74–1.19)	‐0.04 (‐1.5–1.48)	1.16 (‐1.97–4.28)

ACS NSQIP, American College of Surgeons National Surgical Quality Improvement Program; WHODAS, World Health Organization Disability assessment schedule.

In the 168 patients with frailty (CFS ≥ 4), there was an association between frailty and new onset clinically significant disability after 1 year (aOR 3.97, 95%CI 2.54–6.21) and after 30 days (aOR 2.68, 95%CI 1.71–4.21) (Table 6). With regard to continuous WHODAS 2.0 scores, the coefficient of multivariate quantile analysis did not suggest a significant association between frailty and the second quartile (middle score = moderate disability), whereas the coefficient was significant in the first and the third quartile of continuous WHODAS 2.0 scores at 1 year (online Supporting Information Table S9).

**Table 6:** Multivariate logistic regression analysis for the association of frailty with postoperative disability including secondary outcomes. Values are adjusted odds ratio (95%CI).

	New onset clinically significant disability or death	Clinically relevant disability or death	New onset clinically significant disability or death	Clinically relevant disability or death
	At 1 year		At 30 days	
Frailty (CFS ≥ 4)	3.97 (2.54–6.21)	2.65 (1.55–4.54)	2.68 (1.71–4.20)	2.15 (1.25–3.69)
ACS NSQIP*	1.03 (1.01–1.06)	1.03 (1.00–1.06)	1.01 (0.99–1.04)	1.01 (0.98–1.04)
Postoperative complications	1.47 (1.28–1.69)	1.44 (1.23–1.69)	1.47 (1.28–1.69)	1.56 (1.33–1.82)
Cancer surgery	1.23 (0.78–1.95)	1.27 (0.78–2.05)	1.28 (0.81–2.01)	1.42 (0.88–2.28)
Baseline WHODAS 2.0 score	n/a	1.06 (1.04–1.08)	n/a	1.06 (1.04–1.07)

CFS, clinical frailty scale; ACS NSQIP, American College of Surgeons National Surgical Quality Improvement Program; WHODAS, World Health Organization Disability assessment schedule; n/a, not applicable.

* For complications per percent.

Neither OSSS‐3 nor presence of at least one independent household member was associated with new onset clinically significant disability after 1 year or at 30 days in the univariate logistic regression model (1 year: OR 0.87, 95%CI 0.54–1.39 and 0.76, 95%CI 0.47–1.24 for moderate and strong support, respectively; and OR 0.84, 95%CI 0.60–1.18 for at least one independent household member).

Sensitivity analysis limited to survivors aligned with main findings (online Supporting Information Table S10). At 1 year, periprocedural risk (ACS NSQIP) and complications (binary) were associated with WHODAS 2.0 ≥ 25% (aOR 1.03, 95%CI 1.01–1.05 and aOR 1.33, 95%CI 1.19–1.49, respectively). No significant associations with WHODAS 2.0 ≥ 25% were found for most Clavien‐Dindo grades, except 1 and 3b. Evaluation of the association of postoperative binary complications with new onset clinically significant disability after 1 year confirmed the findings (OR 1.42, 95%CI 1.28–1.58). Alternative periprocedural risk scores were also associated with new disability (POSPOM: aOR 1.11, 95%CI 1.07–1.15; CCI: aOR 1.29, 95%CI 1.21–1.38).

Periprocedural risk (ACS NSQIP) and complications of all grades except 3a were independently associated with a 5%‐point change in WHODAS 2.0 (including death) after 1 year. At 30 days association with periprocedural risk (ACS NSQIP) and grade 4 complications were not significant (online Supporting Information Table S11).

Results of ordinal logistic regression (disability as defined for the main model) were consistent with the results above. Changing disability categories into ‘none’, ‘mild’, ‘severe’ and ‘death’ attenuated the association of postoperative complications with postoperative disability (online Supporting Information Table S12). Threshold estimates indicated odds increasing progressively across disability categories in both models.

There was no evidence of a significant interaction between sex and postoperative complications and between regular physical activity and complications for the development of disability at 1 year.

## Discussion

In this prospective multicentre cohort, we have shown that 139/1002 (13.9%) of survivors after non‐cardiac surgery had a new clinically significant disability at 1 year, with a higher incidence found among women. In patients with new clinically significant disability at 1 year, the WHODAS 2.0 domains with the largest changes from baseline score were ‘household responsibilities’, ‘joining community events’ and ‘day‐to‐day work’. After adjustment for periprocedural risk, postoperative complications of any severity were associated with new onset disability and death at 1 year. In addition, pre‐operative frailty was strongly associated with new clinically significant disability but (poor) social support was not.

While the psychometric properties of the 12‐item WHODAS 2.0 have been assessed in surgical patients [4, 23] and setting specific cut‐offs have been proposed [6], evidence on disability incidence 1 year after non‐cardiac surgery remains limited. In a cohort of 4361 surgical patients, 21% showed an increase in clinically significant disability (≥ 5% WHODAS 2.0) at 6 months [6]. Another study (in patients aged ≥ 70 y) reported that up to one in three patients may experience increased disability (≥ 5% change from baseline) 6 months after surgery [24]. In a North American single‐centre cohort of 307 patients aged ≥ 65 y having elective non‐cardiac surgery, 10% of the 183 patients with complete follow‐up (60%) experienced a significant increase in disability at 6 months [25].

Few previous studies have reported on 12‐month disability [4, 7, 26]. In a cohort (n = 510) including 10% cardiac and 10% neurosurgery cases, 13% developed new disability using a 25% cutoff [4]. In a Japanese single‐centre study of 2921 patients aged ≥ 55 y, 10% developed clinically significant disability and 4% died [26]. Wijeysundera et al. investigated 2007 patients aged ≥ 65 y undergoing elective non‐cardiac surgery [7]; one in five patients experienced new disability using the 25% cut off at 1 year, with highest disability rates after 1 month. Our findings extend previous research by providing long‐term data from a large, multicentre German cohort, applying the WHODAS cut‐offs proposed for the surgical setting. We found a 13.9% incidence of new clinically significant disability at 1 year, confirming a substantial burden among survivors of non‐cardiac surgery.

Research on factors linked to new disability after non‐cardiac surgery is limited. Shulman et al. developed and validated a score predicting death or disability at 6 months, based on age, baseline WHODAS 2.0, dementia and chronic kidney disease, showing good accuracy and calibration [24]. Intra‐operative factors such as surgery duration or type did not predict disability. Ida et al. described a large number of pre‐operative factors independently associated with new disability at 12 months, including modifiable factors, such as malnutrition and high BMI; and non‐modifiable factors, including age or intra‐operative blood loss [26]. Recently, Wijeysundera et al. reported prospective data on new disability after 1 year [7]. The authors identified several baseline risk factors, including: frailty; cognitive impairment; mobility aid use; open surgical procedures; and unmet social supports that were linked to postoperative disability. Our analysis also indicated that the ACS NQIP score, which incorporates intra‐operative elements by accounting for the procedure itself, was associated with postoperative disability. Furthermore, our data confirm the independent association between various validated scores of periprocedural risk estimation (CCI, POSPOM and CFS) and new‐onset disability at 1 year. However, these baseline factors are often non‐modifiable, with the potential exception of frailty that may be affected by prehabilitation.

Postoperative complications appear to be relevant and potentially modifiable factors in the development of disability, as suggested by higher disability scores in patients with adverse events after non‐cardiac surgery [27]. While complications were linked to increased disability at 30 days, Shulman et al. found no association with 6‐month disability‐free survival in a cohort of 510 patients [4]. In a larger follow‐up study by the same group, complications were not included in the model and the authors concluded that long‐term disability was influenced more by baseline health than by surgical factors [24].

These findings contrast with the significant association between postoperative complications and 12‐month disability observed in our cohort. Differences in sample size [4], the inclusion of a broader population and a longer follow‐up duration (6 vs. 12 months) may explain these differences. Within the context of the existing literature, our analysis provides an external validation of new onset clinically significant disability using predefined cut‐off values for the peri‐operative setting. Furthermore, within a multicentre prospective cohort, we conducted a complication‐focused analysis and identified additional risk factors associated with postoperative disability. Consistent with these findings, our main multivariable model showed moderate discrimination and acceptable calibration, supporting the reliability of the associations. Common peri‐operative risk scores – including ACS NSQIP, CCI, POSPOM and CFS – were also associated with new‐onset disability at 1 year, suggesting that, beyond their original purpose, they may provide useful information for identifying patients at risk of postoperative functional decline.

Strengths of this study include its prospective, multicentre design with broad inclusion criteria, enhancing generalisability. Our multicentre cohort, derived from eight German university hospitals, is likely similar to other high‐income healthcare systems with comparable peri‐operative structures, case‐mix and standards of surgical and anaesthetic care. However, differences in healthcare organisation, peri‐operative pathways, rehabilitation access and post‐discharge support may limit direct applicability to other populations, particularly ambulatory surgery patients, which is a growing population. The 12‐month follow‐up aligns with StEP recommendations and adds to the limited evidence on long‐term postoperative disability. Using a setting‐specific cut‐off, stratifying incidence by sex and reporting item‐level WHODAS 2.0 data offers a detailed picture of disability after non‐cardiac surgery. A comprehensive assessment of peri‐operative factors, along with the evaluation of complications – a potentially modifiable risk factor – provides valuable insights into determinants of postoperative disability.

The main limitation of our study is the 24.7% loss to follow‐up (359/1450) (online Supporting Information Figure S1), although this is comparable to similar studies (14–41%) [4, 25, 26]. Despite repeated attempts to contact patients by telephone and postal mail, several patients could not be reached, and unfortunately detailed reasons for non‐response are not available. Despite the loss of patients in follow‐up, we do not consider it to have introduced major selection bias since the baseline characteristics of patients without follow‐up presented only minor differences compared with those included in the 12‐month analysis (online Supporting Information Table S2).

Additional limitations include that the conduct of the study in university hospitals may limit generalisability due to differences in patient populations and care settings. Furthermore, patients with insufficient proficiency in the study language were not included, leading to potential selection bias which may limit external validity. Although patients with reading difficulties were not omitted and, where feasible, completed the study in an interviewer‐assisted format consistent with the validated telephone administration, such support was contingent on available time and personnel resources and may not have been implementable uniformly. Furthermore, a nested cohort focusing on postoperative delirium required separate consent and multiple visits, which likely reduced participation. Consequently, low enrolment in this sub‐analysis, together with the small number of specific complications, prevented meaningful assessment of delirium and individual complications.

In conclusion, we found that 1 year after non‐cardiac surgery, 14% of surviving patients experienced new‐onset clinically significant disability. Pre‐operative frailty was strongly associated, whereas social support was not. Even mild postoperative complications were consistently associated with new disability. This underscores the importance of early detection and management of complications to mitigate adverse functional outcomes.

## Supporting information


**Appendix S1.** PACORUS‐D Investigators.

**Figure S1.** Scores for the single items at 1 year in patients with new onset disability after 1 year stratified by sex.
**Figure S2.** Patient‐reported disability from pre‐operative status to 1 year after non‐cardiac surgery stratified by ACS NSQIP score quartiles.
**Figure S3.** Patient‐reported disability from pre‐operative status to 1 year after non‐cardiac surgery stratified by surgical category.

**Table S1.** Full baseline patient characteristics.
**Table S2.** Characteristics and surgical information of patients with and without new onset clinically significant disability at 1 year as well as patients without any disability at 1 year, patients with 1 year mortality and patients lost to follow up at 1 year.
**Table S3.** Disability after non‐cardiac surgery.
**Table S4.** Details of type of surgery.
**Table S5.** Patient‐reported disability at 30 days and 1 year by surgical category.
**Table S6.** Patient‐reported disability at 30 days and 1 year by ACS NSQIP scores and postoperative complications.
**Table S7.** Documented adverse events of patients with and without primary endpoint and corresponding relative risk.
**Table S8.** Multivariate quantile regression analysis for the association of periprocedural risk and postoperative complications with continuous WHODAS 2.0 scores after 30 days.
**Table S9.** Multivariate quantile regression analysis for the association of frailty with continuous WHODAS 2.0 score after 30 days and 1 year.
**Table S10.** Multivariate logistic regression analysis for the association of pre‐operative risk and postoperative complications with postoperative disability at 30 days and 1 year including only survivors.
**Table S11.** Multivariate logistic regression model for the association of pre‐operative risk and postoperative complications with 5%‐point change (including death) of WHODAS 2.0 score after 30 days and 1 year.
**Table S12.** Multivariate ordinal regression model for the association of pre‐operative risk and postoperative complications with postoperative disability.
