# Supplementary material for: Disability after non‐cardiac surgery – patient‐centred outcomes and resource utilisation after non‐cardiac surgery (PACORUS): a prospective multicentre cohort study*
Source: Anaesthesia. 2026 Jun 11;81(11):1497–509. doi: 10.1111/anae.70248 (PMC13659787; doi:10.1111/anae.70248)
Supplement: Supplementary file 1 [file ANAE-81-1497-s001.docx]

**Table S1**. Full baseline patient characteristics. Values are median (IQR [range]) or number (proportion).

|  | **Full dataset**  **n = 1081** | **Patients with new onset clinically significant disability or dead at 1 year**  **n = 218** | **Patients with no new onset clinically significant disability and alive at 1 year**  **n = 863** |
| --- | --- | --- | --- |
| Age; y | 67 (60-75 [50-101]) | 71 (62-78 [50-94]) | 66 (59-74 [50-101]) |
| 50-65 | 457 (42.3%) | 68 (31.2%) | 389 (45.1%) |
| 65-74 | 351 (32.5%) | 69 (31.7%) | 282 (32.7%) |
| ≥ 75 | 273 (25.2%) | 81 (37.2%) | 192 (22.2%) |
| Male | 726 (67.1%) | 130 (59.6%) | 596 (69.1%) |
| Oslo Social Support-3 scale | n = 1080 | n = 217 | n = 863 |
| Poor social support | 121 (11.2%) | 28 (12.9%) | 93 (10.8%) |
| Moderate social sport | 493 (45.6%) | 102 (47.0%) | 391 (45.3%) |
| Strong social support | 466 (43.2%) | 87 (40.1%) | 379 (43.9%) |
| Pre-operative Clinical Frailty Scale | n = 834 | n = 177 | n = 657 |
| Very fit | 90 (10.8%) | 6 (3.4%) | 84 (12.8%) |
| Well | 315 (37.8%) | 38 (21.5%) | 277 (42.2%) |
| Managing well | 229 (27.5%) | 51 (28.8%) | 178 (27.1%) |
| Vulnerable | 98 (11.8%) | 41 (23.2%) | 57 (8.7%) |
| Mildly frail | 43 (5.2%) | 16 (9.0%) | 27 (4.1%) |
| Moderately frail | 32 (3.8%) | 14 (7.9%) | 18 (2.7%) |
| Severely frail | 23 (2.8%) | 11 (6.2%) | 12 (1.8%) |
| Very severely frail | 4 (0.3%) | 0 (0.0%) | 4 (0.6%) |
| Pre-operative functional status | n = 1080 | n = 218 | n = 863 |
| Fully independent | 905 (83.8%) | 144 (66.1%) | 761 (88.1%) |
| Partially dependent | 164 (15.2%) | 69 (31.7%) | 95 (11.0%) |
| Fully dependent | 12 (1.1%) | 5 (2.2%) | 7 (0.9%) |
| Baseline WHODAS 2.0 score | 10.4 (4.2-27.1 [0.0-85.4]) | 23 (10.4-40 [0.0-72.9]) | 8.3 (4.2-23 [0.0-85.4]) |
| ASA physical status | N=1081 | N=218 | N=863 |
| 1–2 | 573 (53.0%) | 76 (34.9%) | 497 (57.6%) |
| ≥ 3 | 508 (47.0%) | 142 (65.1%) | 366 (42.4%) |
| Diabetes mellitus | 185 (17.1%) | 51 (23.4%) | 134 (15.5%) |
| Cancer diagnosis | 480 (44.4%) | 121 (55.6%) | 359 (41.6%) |
| Hemiplegia | 8 (0.7%) | 1 (0.5%) | 7 (0.8%) |
| Dementia | 1 (0.1%) | 0.0 (0%) | 1 (0.1%) |
| Chronic obstructive pulmonary disease | 74 (6.8%) | 25 (11.5%) | 49 (5.7%) |
| Chronic heart failure | 144 (13.3%) | 46 (21.1%) | 98 (11.4%) |
| Ischemic heart disease | 139 (12.9%) | 39 (17.9%) | 100 (11.6%) |
| History of myocardial infarction | 62 (5.7%) | 13 (6.0%) | 49 (5.7%) |
| Cardiac arrhythmia | 137 (12.7%) | 36 (16.5%) | 101 (11.7%) |
| Peripheral artery disease | 231 (21.3%) | 68 (31.2%) | 163 (18.9%) |
| Carotid artery disease | 97 (9.0%) | 26 (11.9%) | 71 (8.2%) |
| Smoker (n = 1077) | 193 (17.9%) | 45 (20.6%) | 148 (17.1%) |
| Alcohol abuse (n = 1076) | 57 (5.3%) | 8 (3.7%) | 49 (5.7%) |
| Renal disease | 139 (12.9%) | 44 (20.2%) | 95 (11.0%) |
| Liver disease | 80 (7.4%) | 18 (8.3%) | 62 (7.2%) |
| Daily physical activities | n = 896 | n = 173 | n = 723 |
| Inactive | 173 (19.3%) | 42 (24.3%) | 131 (18.1%) |
| Regularly low level | 199 (22.3%) | 55 (31.8%) | 144 (19.9%) |
| 20–60 min per week | 160 (17.7%) | 30 (17.3%) | 130 (18.0%) |
| 1–3 h per week | 177 (19.8%) | 24 (13.9%) | 153 (21.2%) |
| > 3 h per week | 187 (20.9%) | 22 (12.7%) | 165 (22.8%) |

**Table S2:** Characteristics and (post-)surgical information of patients with and without “new onset clinically significant disability” at 1 year, as well as patients without any disability at 1 year, patients with 1 year mortality and patients lost to follow up at 1 year.

|  | **Patients with “new onset clinically significant disability” at 1 year n (%)** | **Patients without “new onset clinically significant disability” at 1 year n (%)** | **Patients with 1 year mortality** | **Patients with incomplete follow up after 1 year (missing data about mortality/WHODAS 2.0) n (%)** |
| --- | --- | --- | --- | --- |
|  | 139/1081 (12.9%) | 942/1081 (87.1%) | 79/1081 (7.3%) | 359/1450 (24.7%) |
| **Age (years) median (Q1-Q3 [range])** | 71 (62-77 [50-88]) | 67 (59-74 [50-101]) | 74 (62-79 [52-94]) | 65 (58-75 [50-92]) |
| 50-65 | 43 (30.9%) | 414 (44.0%) | 25 (31.6%) | 168 (46.8%) |
| 65-74 | 51 (36.7%) | 300 (31.8%) | 18 (22.8%) | 97 (27.0%) |
| ≥75 | 45 (32.4%) | 228 (24.2%) | 36 (45.6%) | 94 (26.2%) |
| **Male sex** | 86 (61.9%) | 640 (67.9%) | 44 (5.6%) | 227 (63.2%) |
| **OSS-3 scale** | N=138 | N=942 | N=79 | N=359 |
| Poor social support | 19 (13.8%) | 102 (10.8%) | 9 (11.3%) | 65 (18.2%) |
| Moderate social support | 63 (45.7%) | 430 (45.6%) | 39 (49.4%) | 147 (40.9%) |
| Strong social support | 56 (40.5%) | 410 (43.5%) | 31 (39.3%) | 147 (40.9%) |
| **Preoperative** **frailty** | N=108 | N=726 | N=69 | N=240 |
| Very fit | 5 (4.6%) | 85 (11.7%) | 1 (1.5%) | 11 (4.5%) |
| Well | 27 (25.0%) | 288 (39.7%) | 11 (15.9%) | 80 (33.3%) |
| Managing well | 30 (27.8%) | 199 (27.4%) | 21 (30.5%) | 66 (27.5%) |
| Vulnerable | 25 (23.2%) | 73 (10.1%) | 16 (23.2%) | 40 (16.6%) |
| Mildly frail | 10 (9.2%) | 33 (4.5%) | 6 (8.7%) | 22 (9.3%) |
| Moderately frail | 7 (6.5%) | 25 (3.4%) | 7 (10.1%) | 12 (5.0%) |
| Severely frail | 4 (3.7%) | 19 (2.6%) | 7 (10.1%) | 8 (3.4%) |
| Very severely frail | 0 (0.0%) | 4 (0.6%) | 0 (0.0%) | 1 (0.4%) |
| **Preoperative functional status** | N=139 | N=942 | N=79 | N=359 |
| Fully independent | 93 (66.9%) | 812 (86.2%) | 51 (64.6%) | 296 (82.5%) |
| Partially dependent | 45 (32.4%) | 119 (12.6%) | 24 (30.4%) | 57 (15.8%) |
| Fully dependent | 1 (0.7%) | 11 (1.2%) | 4 (5.0%) | 6 (1.7%) |
| **ASA physical status** | N=139 | N=942 | N=79 | N=359 |
| I-II | 55 (39.6%) | 518 (54.9%) | 21 (26.6%) | 182 (50.7%) |
| >=III | 84 (60.4%) | 424 (45.1%) | 58 (73.4%) | 177 (49.3%) |
| **Diabetes mellitus** | 37 (26.6%) | 148 (15.7%) | 14 (17.7%) | 82 (22.8%) |
| **Cancer diagnosis** | 65 (46.8%) | 415 (44.1%) | 56 (70.9%) | 157 (43.7%) |
| **Hemiplegia** | 1 (0.7%) | 7 (0.7%) | 0 (0.0%) | 7 (1.9%) |
| **Dementia** | 0 (0.0%) | 1 (0.1%) | 0 (0.0%) | 3 (0.8%) |
| **COPD** | 11 (7.9%) | 63 (6.7%) | 14 (17.7%) | 36 (10.0%) |
| **Chronic heart failure** | 29 (20.8%) | 115 (12.2%) | 17 (21.5%) | 83 (23.1%) |
| **Ischemic heart disease** | 26 (18.7%) | 113 (11.9%) | 13 (16.4%) | 60 (16.7%) |
| **History of myocardial infarction** | 10 (7.2%) | 52 (5.5%) | 3 (3.8%) | 22 (6.1%) |
| **Cardiac arrythmia** | 21 (15.1%) | 116 (12.3%) | 15 (18.9%) | 53 (14.8%) |
| **PAD** | 38 (27.3%) | 193 (20.5%) | 30 (37.9%) | 94 (26.2%) |
| **CAD** | 15 (10.8%) | 82 (8.7%) | 11 (13.9%) | 41 (11.4%) |
| **Smoker (n=1077)** | 30 (21.6%) | 163 (17.3%) | 15 (19.0%) | 77 (21.4%) |
| **Alcohol abuse (n=1076)** | 7 (5.0%) | 50 (5.3%) | 1 (1.3%) | 22 (6.1%) |
| **Renal disease** | 23 (16.5%) | 116 (12.3%) | 21 (26.5%) | 66 (18.4%) |
| **Liver disease** | 6 (4.3%) | 74 (7.9%) | 12 (15.1%) | 30 (8.4%) |
| **Daily physical activities** | n=104 | n=792 | N=69 | n=197 |
| Inactive | 25 (24.1%) | 148 (18.7%) | 17 (24.6%) | 51 (25.9%) |
| regularly low level | 32 (30.7%) | 167 (21.1%) | 23 (33.4%) | 49 (24.8%) |
| 20-60 minutes/week | 16 (15.4%) | 144 (18.2%) | 14 (20.2%) | 49 (24.8%) |
| 1-3 hours/week | 16 (15.4%) | 161 (20.3%) | 8 (11.7%) | 28 (14.2%) |
| >3hours/week | 15 (14.4%) | 172 (21.7%) | 7 (10.1%) | 20 (10.2%) |
|  |  |  |  |  |
| ***Preoperative risk scores*** |  |  |  |  |
| **ACS-NSQIP complication (%, median (Q1-Q3 [range])** | 8.6 (5.4-16.9 [1.4-44.9]) | 6.7 (4.4-12.8 [0.0-52.2]) | 15.8 (8.1-22.8 [1.4-52.2]) | 7.6 (4.3-13.7 [0.0-38.6]) |
| **ACS-NSQIP death (%, median (Q1-Q3 [range])** | 0.6 (0.1-2.0 [0.0-10.6]) | 0.2 (0.0-1.0 [0.0-46.6]) | 1.6 (0.3-5.1 [0-46.6]) | 0.3 (0.1-1.1 [0.0-26.6]) |
| **POSPOM (points, median (Q1-Q3 [range])** | 28 (25-32 [14-42]) | 26 (21-29 [7-44]) | 30 (27-33 [14-44]) | 27 (23-31 [12-44]) |
| **Charlson Comorbidity Index (points, median (Q1-Q3 [range])** | 5 (4-7 [1-13]) | 4 (3-6 [1-13]) | 7 (5-9 [1-13]) | 5 (3-7 [1-15]) |
|  |  |  |  |  |
| ***Procedural factors*** |  |  |  |  |
| **Emergency case** | 11 (7.9%) | 63 (6.7%) | 8 (10.1%) | 33 (9.2%) |
| **Major surgery (n=1076)** | 73 (53.5%) | 504 (53.5%) | 55 (69.6%) | 181 (50.4%) |
| **Cancer surgery** | 43 (30.9%) | 341 (36.2%) | 47 (59.4%) | 129 (35.9%) |
| ***Postoperative factors*** |  |  |  |  |
| **ICU admission** | 29 (20.8%) | 130 (13.8%) | 35 (44.3%) | 60 (16.7%) |
| **Unplanned ICU admission** | 8/29 (27.5%) | 24/130 (18.5%) | 10/35 (28.5%) | 17/60 (28.3%) |
| **Postoperative complication** | 59 (42.4%) | 249 (26.4%) | 48 (60.8%) | n=135 (37.6%) |
| **Clavien Dindo Grade** |  |  |  |  |
| I | 18 (30.5%) | 86 (34.5%) | 9 (18.8%) | 52 (38.5%) |
| II | 18 (30.5%) | 76 (30.5%) | 9 (18.8%) | 34 (25.2%) |
| IIIa | 6 (10.2%) | 26 (10.4%) | 5 (10.4%) | 12 (8.9%) |
| IIIb | 9 (15.2%) | 30 (12.2%) | 5 (10.4%) | 29 (21.5%) |
| IVa | 8 (13.6%) | 10 (4.0%) | 2 (4.2%) | 8 (5.9%) |
| IVb | 0 (0.0%) | 3 (1.2%) | 1 (2.0%) | 0 (0.0%) |
| V | 0 (0.0%) | 18 (7.2%) | 17 (35.4%) | 0 (0.0%) |
| **In-hospital death** | 0 (0.0%) | 18 (1.9%) | 18 (22.7%) | 0 (0.0%) |
| **30 days mortality** | 0 (0.0%) | 14 (1.5%) | 14 (17.7%) | 0 (0.0%) |
| **1 year mortality** | 0 (0.0%) | 79 (8.3%) | 79 (100%) | n/a |
| **WHDOAS-2.0 Score (%, median (Q1-Q3 [range])** |  |  |  |  |
| Baseline | 25 (12.5-39.6 [0.0-72.9]) | 10.4 (4.2-23 [0.0-85.4]) | 18.8 (8.3-43.8 [0.0-72.9]) | 14.6 (6.3-35.4 [0-83.3]) |
| 30 days | 39.6 (24-58.3 [4.2-100.0]) | 16.6 (4.2-35.4 [0.0-100.0]) (n=901) | 43.8 (14.6-75 [0.0-100.0])  (n=66) | 25 (10.4-45.8 [0.0-100.0]) |
| 1 year | 52.1 (41.7-70.8 [35.4-100.0]) | 8.3 (2.1-23 [0.0-83.3]) | n/a | n/a |

ACS-NSQIP: American College of Surgeons-National Surgical Quality Improvement Program, ASA: American Society of Anesthesiologists, CAD : carotid artery disease, COPD : chronic obstructive pulmonary disease, ICU: intensive care unit, OSS : Oslo social support, PAD : peripheral artery disease, POSPOM: Preoperative Score to Predict Postoperative Mortality, WHODAS : World Health Organization Disability assessment schedule,

**Table S3:** Disability after non-cardiac surgery

|  | 1 year  n=1081 (100%) | 30 days  n=1066 (100%) |
| --- | --- | --- |
| No disability (WHODAS 2.0 ≤ 15%) | 623 (57.6%) | 468 (43.9%) |
| Mild disability (WHODAS 2.0 16-34%) | 172 (5.9%) | 263 (24.7%) |
| Significant disability (WHDOAS 2.0 ≥ 35%) | 207 (19.1%) | 321 (30.1%) |
| **“****New onset clinically significant disability” (WHODAS 2.0 ≥ 35% and increase ≥ 5%)** | **139 (12.9%)** | **238 (22.3%)** |
| Dead | 79 (7.3%) | 14 (1.3%) |

WHODAS: World Health Organization Disability assessment schedule

**Table S4**: Details of type of surgery. Surgical categories have been documented according to POSPOM Score. The classification into minor and major surgery was performed by two independent clinicians. Minor surgery: orthopaedic other, endoscopic digestive, gastrointestinal minor, hepatic minor, vascular minor, urologic minor; Major surgery: arthroplasty and spine, orthopaedic trauma (hip/femur fractures), multiple trauma-related, gastrointestinal major, hepatic major, renal transplant, transplant, vascular major, urologic major, thoracic

|  | **Full cohort (analysed at 1 year) n=1076/1081** | **Patients analysed at 30 days n= 1061/1066** |
| --- | --- | --- |
| **Major surgery** | 577 (53.6%) | 561 (52.8%) |
| Arthroplasty and Spine | 72 (6.7%) | 65 (6.1%) |
| Orthopaedic trauma (hip or femur fractures) | 30 (2.8%) | 30 (2.8%) |
| multiple-trauma related | 6 (0.7%) | 7 (0.7%) |
| gastrointestinal major | 183 (17.0%) | 177 (16.7%) |
| hepatic major | 22 (2.0%) | 24 (2.3%) |
| renal transplant | 13 (1.2%) | 14 (1.3%) |
| vascular major | 74 (6.9%) | 68 (6.4%) |
| urologic major | 145 (13.5%) | 144 (13.6%) |
| thoracic | 32 (3.0%) | 32 (3.0%) |
| **Minor surgery** | 499 (46.4%) | 500 (47.1%) |
| orthopaedic other | 109 (10.1%) | 109 (10.3%) |
| endoscopic digestive | 10 (0.9%) | 10 (0.9%) |
| gastrointestinal minor | 89 (8.2%) | 88 (8.3%) |
| hepatic minor | 3 (0.3%) | 3 (0.3%) |
| vascular minor | 92 (8,5%) | 94 (8.9%) |
| urologic minor | 196 (18.2%) | 196 (18.5%) |

**Table S5:** Patient-reported disability at 1 year and 30 days by surgical category

|  | **“New onset clinically significant disability” at 1 year n (%)** | **Death at 1 year n (%)** | **“New onset clinically significant disability” at 30 days n (%)** | **Death at 30 days (n%)** |
| --- | --- | --- | --- | --- |
| **Major surgery** | 73/577 (12.7%) | 55/577 (9.5%) | 163/561 (29.1%) | 9/561 (1.6%) |
| Arthroplasty and Spine | 6/72 (8.3%) | 4/72 (5.5%) | 19/65 (29.2%) | 0/65 (0.0%) |
| Orthopaedic trauma (hip or femur fractures) | 10/30 (33.3%) | 1/30 (3.3%) | 17/30 (56.7%) | 0/30 (0.0%) |
| multiple-trauma related | 1/6 (16.7%) | 0/6 (0.0%) | 4/7 (57.1%) | 0/7 (0.0%) |
| gastrointestinal major | 26/183 (14.2%) | 28/183 (15.3%) | 64/177 (36.2%) | 6/177 (3.4%) |
| hepatic major | 2/22 (9.1%) | 3/22 (13.6%) | 3/24 (12.5%) | 0/24 (0.0%) |
| renal transplant | 1/13 (7.7%) | 1/13 (7.7%) | 4/14 (28.6%) | 0/14 (0.0%) |
| vascular major | 12/74 (16.2%) | 7/74 (9.5%) | 15/68 (22.1%) | 2/68 (2.9%) |
| urologic major | 9/145 (6.2%) | 7/145 (4.8%) | 28/144 (19.4%) | 1/144 (0.7%) |
| thoracic | 6/32 (18.8%) | 4/32 (12.5%) | 9/32 (28.1%) | 0/32 (0.0%) |
| **Minor surgery** | 66/499 (13.2%) | 24/499 (4.8%) | 75/500 (15.0%) | 5/500 (1.0%) |
| orthopaedic other | 11/109 (10.1%) | 5/109 (4.6%) | 35/109 (32.1%) | 2/109 (1.8%) |
| endoscopic digestive | 0/10 (0.0%) | 0/10 (0.0%) | 2/10 (20.0%) | 0/10 (0.0%) |
| gastrointestinal minor | 17/89 (19.1%) | 5/89 (5.6%) | 18/88 (20.5%) | 1/88 (1.1%) |
| hepatic minor | 0/3 (0.0%) | 0/3 (0.0%) | 0/3 (0.0%) | 0/3 (0.0%) |
| vascular minor | 15/92 (16.3%) | 4/92 (4.3%) | 8/94 (8.5%) | 2 /94 (2.1%) |
| urologic minor | 23/196 (11.7%) | 10/196 (5.1%) | 12/196 (6.1%) | 0/196 (0.0%) |

**Table S6**: Patient-reported disability at 1 year and 30 days by ACS-NSQIP scores for postoperative complications (quartiles) and by postoperative complications (defined by Clavien-Dindo)

|  | **“New onset clinically significant disability” at 1 year n (%)** | **“New onset clinically significant disability”^1^ at 30 days n (%)** |
| --- | --- | --- |
| ACS-NSQIP* ≤ 4.5% | 19/273 (6.9%) | 39/260 (15.0%) |
| ACS-NSQIP 4.5-7.0% | 38/282 (13.5%) | 55/282 (19.5%) |
| ACS-NSQIP 7.0-13.3% | 37/258 (14.3%) | 66/260 (25.4%) |
| ACS-NSQIP > 13.3% | 45/268 (16.8%) | 78/263 (29.7%) |
| Complication grade I | 18/104 (17.3%) | 35/105 (33.3%) |
| Complication grade II | 18/94 (19.1%) | 25/93 (26.9%) |
| Complication grade IIIa | 6/32 (18.8%) | 13/32 (40.6%) |
| Complication grade IIIb | 9/39 (23.1%) | 19/37 (51.4%) |
| Complication grade IVa/b | 8/22 (36.4%) | 10/22 (45.5%) |

ACS-NSQIP: American College of Surgeons-National Surgical Quality Improvement Program

**Table S7:** Documented adverse events of patients with and without primary endpoint and corresponding relative risk

|  | **Full dataset** | **Patients with primary endpoint: “new onset clinically significant disability” or dead at 1 year** | **Patients without primary endpoint: no “new onset clinically significant disability” and alive at 1 year** | **Relative risk (96%CI)** |
| --- | --- | --- | --- | --- |
|  | 1081 (100%) | 218/1081 (20.2%) | 863/1081 (79.8%) |  |
| Cardiac arrest | 8 (0.7%) | 8 (3.6%) | 0 (0.0%) | 67.07 (3.89-1157.57) |
| Myocardial infarction | 0 (0.0%) | 0 (0.0%) | 0 (0.0%) | 3.95 (0.08-198.28) |
| Stroke | 2 (0.2%) | 1 (0.5%) | 1 (0.1%) | 3.96 (0.25-63.04) |
| Symptomatic heart failure | 7 (0.6%) | 6 (2.7%) | 1 (0.1%) | 23.75 (2.87-196.27) |
| Pulmonary embolism | 5 (0.5%) | 3 (1.4%) | 2 (0.2%) | 5.94 (1.00-35.32) |
| Pneumonia | 18 (1.6%) | 11 (5.0%) | 7 (0.8%) | 6.22 (2.44-15.86) |
| Respiratory failure | 17 (1.6%) | 12 (5.5%) | 5 (0.6%) | 9.50 (3.38-26.28) |
| Delirium | 13 (1.2%) | 8 (3.7%) | 5 (0.6%) | 6.33 (2.09-19.17) |
| Acute kidney disease | 17 (1.6%) | 12 (5.5%) | 5 (0.6%) | 9.50 (3.38-26.68) |
| Surgical site infection | 39 (3.6%) | 15 (6.9%) | 24 (2.7%) | 2.47 (1.32-4.64) |
| Bleeding | 26 (2.4%) | 13 (5.9.%) | 13 (1.5%) | 3.96 (1.86-8.42) |
| Anemia | 60 (5.6%) | 27 (12.4%) | 33 (3.8%) | 3.24 (1.99-5.27) |

**Table S8:** Multivariate quantile regression analysis for the association of periprocedural risk and postoperative complications (ACS-NSQIP score for postoperative complications) with continuous WHODAS 2.0 scores (%) after 30 days

| Parameter | **0.25-quantile**  **adjusted regression coefficient (95%CI)** | **0.5-quantile**  **adjusted regression coefficient (95%CI)** | **0.75-quantile**  **adjusted regression coefficient (95%CI)** |
| --- | --- | --- | --- |
| ACS NSQIP | 0.18 (0.08-0.28) | 0.12 (-0.03-0.28) | 0.21 (-0.10-0.51) |
| Postoperative Complication | 1.04 (0.47-1.62) | 2.44 (1.52-3.35) | 5.71 (3.92-7.50) |
| WHODAS 2.0 score at baseline | 0.64 (0.61-0.69) | 0.85 (0.79-0.90) | 0.83 (0.72-0.94) |
| Cancer surgery | 0.48 (-0.97-1.92) | 1.58 (-0.73-3.39) | 4.66 (0.16-9.15) |

ACS-NSQIP: American College of Surgeons-National Surgical Quality Improvement Program, WHODAS: World Health Organization Disability assessment schedule

**Table S9:** Multivariate quantile regression analysis for the association of frailty (CFS≥4) with continuous WHODAS 2.0 score (%) after 1 year and 30 days:

| Parameter | **0.25-quantile**  **adjusted regression coefficient (95%CI)** | **0.5-quantile**  **adjusted regression coefficient (95%CI)** | **0.75-quantile**  **adjusted regression coefficient (95%CI)** |
| --- | --- | --- | --- |
| 1 year | 3.26 (1.02-5.51) | 2.49 (-0.48-5.46) | 8.02 (0.61-15.43) |
| 30 days | 0.04 (-2.33-2.42) | 3.47 (0.08-6.87) | 11.29 (2.66-19.92) |

**Table S10:** Multivariate logistic regression analysis for the association of preoperative risk (ACS-NSQIP score for postoperative complications per percent) and postoperative complications (defined by Clavien-Dindo) with postoperative disability at 30 days and 1 year including only survivors

|  | **“New onset clinically significant disability” at 1 year** | **“New onset clinically significant disability” at 30 days** |
| --- | --- | --- |
| ACS NSQIP | 1.04 1.01-1.06) | 1.02 (1.00-1.04) |
| Complication grade I | 1.80 (1.02-3.20) | 2.17 (1.38-3.41) |
| Complication grade II | 1.94 (1.07-3.51) | 1.50 (0.90-2.51) |
| Complication grade IIIa | 2.11 (0.81-5.49) | 2.72 (1.29-5.76) |
| Complication grade IIIb | 2.53 (1.12-5.71) | 4.33 (2.18-8.57) |
| Complication grade IVa/b | 5.14 (1.92-13.76) | 3.40 (1.38-8.42) |
| Cancer surgery | 0.64 (0.42-0.97) | 1.06 (0.77-1.46) |

ACS-NSQIP: American College of Surgeons-National Surgical Quality Improvement Program

**Table S11:** Multivariate logistic regression model for the association of preoperative risk (ACS-NSQIP score for postoperative complications per percent) and postoperative complications (defined by Clavien-Dindo) with 5%-point change (including death) of WHODAS 2.0 score after 1 year and 30 days; main exposures: Risk by ACS NSQIP score, complications by Clavien Dindo; confounder: cancer surgery. The Hosmer–Lemeshow goodness-of-fit test indicated adequate model fit (p = 0.27 for 1 year and p=0.49 for 30 days)

|  | **OR (95%CI) for “5% point change in WHODAS” at 1 year** | **OR (95%CI) for “5% point change in WHODAS” at 30 days** |
| --- | --- | --- |
| ACS NSQIP | 1.03 (1.01-1.05) | 1.01 (0.99-1.03) |
| Complication grade I | 1.56 (1.01-2.40) | 1.83 (1.21-2.78) |
| Complication grade II | 1.92 (1.22-3.02) | 1.62 (1.04-2.54) |
| Complication grade IIIa | 1.69 (0.81-3.54) | 2.97 (1.37-6.46) |
| Complication grade IIIb | 1.99 (1.02-3.86) | 2.08 (1.05-4.13) |
| Complication grade IVa/b | 3.06 (1.26-7.43) | 2.21 (0.90-5.46) |
| Cancer surgery | 1.21 (0.91-1.60) | 1.54 (1.18-2.01) |

ACS-NSQIP: American College of Surgeons-National Surgical Quality Improvement Program

**Table S12:** Multivariate ordinal regression model for the association of preoperative risk (ACS-NSQIP score for postoperative complications per percent) and postoperative complications (defined by Clavien-Dindo) with postoperative disability, categorized as (1) alive and no “new onset clinically significant disability” , “new onset clinically significant disability” (WHODAS 2.0 ≥ 35% and increase ≥ 5%), “dead” and (2) “no disability (WHODAS 2.0 <16%), “mild disability” (WHODAS 2.0 16-34%), “significant disability” (WHODAS 2.0 ≥16%), “death” after 1 year; main exposures: Risk by ACS NSQIP score, complications by Clavien Dindo; confounder: cancer surgery.

|  | **OR (95%CI) for disability (1) at 1 year** | **OR (95%CI) for disability (2) at 1 year** |
| --- | --- | --- |
| ACS NSQIP | 1.05 (1.03-1.07) | 1.06 (1.04-1.07) |
| Complication grade I | 1.79 (1.10-2.92) | 1.44 (0.97-2.15) |
| Complication grade II | 1.78 (1.07-2.97) | 1.20 (0.78-1.85) |
| Complication grade IIIa | 2.47 (1.14-5.35) | 1.55 (0.75-3.23) |
| Complication grade IIIb | 2.55 (1.27-5.09) | 2.46 (1.37-4.45) |
| Complication grade IVa/b | 3.37 (1.46-7.80) | 2.43 (1.08-5.47) |
| Cancer surgery | 1.11 (0.79-1.54) | 0.82 (0.63-1.06) |

ACS-NSQIP: American College of Surgeons-National Surgical Quality Improvement Program
