# Supplementary material for: Disability after non‐cardiac surgery – patient‐centred outcomes and resource utilisation after non‐cardiac surgery (PACORUS): a prospective multicentre cohort study*
Source: Anaesthesia. 2026 Jun 11;81(11):1497–509. doi: 10.1111/anae.70248 (PMC13659787; doi:10.1111/anae.70248)
Supplement: Supplementary file 2 [file ANAE-81-1497-s002.docx]

**Appendix S1.** PACORUS Investigators:

Düsseldorf:

- Onur Aydemir
- Tabea Schüssler

Aachen:

- Linda Grüßer

Bonn:

- Ana Kowark
- Andrea Kunsorg
- Maria Wittmann

Köln:

- Franziska Torabi
- Jakob Labus

Essen:

- Jens Brands
- Thorsten Brenner
- Theresa Berger

Heidelberg:

- Sarah Dehne
- Melanie Etheredge
- Markus A. Weigand

München:

- Simon Schäfer

Leipzig:

- Christian Schlegel
- Antje Seidel
