# Supplementary material for: Disability after non‐cardiac surgery – patient‐centred outcomes and resource utilisation after non‐cardiac surgery (PACORUS): a prospective multicentre cohort study*
Source: Anaesthesia. 2026 Jun 11;81(11):1497–509. doi: 10.1111/anae.70248 (PMC13659787; doi:10.1111/anae.70248)
Supplement: Supplementary file 3 [file ANAE-81-1497-s003.docx]

**Figure S1**: Scores for the single items at baseline and at 1 year in the patients with new onset disability after 1 year stratified by sex 0, none (green); 1, mild (blue); 2, moderate (yellow); 3, severe (purple); 4, extreme or cannot do (red). Black diamonds, median of the responses.

**Figure S2**: Patient-reported disability from pre-operative status to 1 year after non-cardiac surgery stratified by surgical category.

**Figure S3:** Patient-reported disability from pre-operative status to 1 year after non-cardiac surgery stratified by ACS-NSQIP score quartiles.
